# Effects of rumen-degradable-to-undegradable protein ratio in ruminant diet on *in vitro* digestibility, rumen fermentation, and microbial protein synthesis

**DOI:** 10.14202/vetworld.2021.640-648

**Published:** 2021-03-17

**Authors:** Ezi Masdia Putri, Mardiati Zain, Lili Warly, Hermon Hermon

**Affiliations:** Department of Animal Nutrition, Faculty of Animal Science Andalas University, Kampus Limau Manis, Padang, West Sumatera, Indonesia

**Keywords:** digestibility, microbial protein synthesis, protein, rumen characteristic, rumen degradable protein, rumen undegradable protein

## Abstract

**Background and Aim:**

Feeding ruminants must notice the degradability of feed, especially protein. Microbial rumen requires ammonia from rumen degradable protein (RDP) beside that ruminant require bypass protein or rumen undegradable protein (RUP) and microbial crude protein. The aim of the study was to discover the best RDP:RUP ratio in beef cattle diets commonly used by Indonesian farmers using an *in vitro* methodology.

**Materials and Methods:**

Samples of *Pennisetum purpureum*, *Leucaena leucocephala*, *Indigofera zollingeriana*, cassava, maize, palm kernel cake, rice bran, and tofu waste were formulated into dietary treatments (dry matter [DM] basis). All experiments were carried out using a 3×3×2 factorial, randomized block design with three replications. Treatments consisted of three protein levels (12%, 14%, and 16%), two energy levels (65% and 70%), and three RDP:RUP ratio levels (55:45, 60:40, and 65:35). The experimental diets were incubated *in vitro* using buffered rumen fluid for 48 h at 39°C. After incubation, the supernatants were analyzed to determine pH, ammonia concentration, total volatile fatty acid (VFA), and microbial protein synthesis. The residues were analyzed to determine DM, organic matter, protein, and RUP digestibility.

**Results:**

Increased protein, energy, and RDP levels increased digestibility, ammonia concentrations, total VFAs, and microbial protein synthesis (p<0.05), while rations with 16% protein lowered these parameters (p<0.05).

**Conclusion:**

Increased dietary protein (from 12% to 14% DM), energy (from 65% to 70% DM), and RDP (from 55% to 65% crude protein [CP]) levels increased nutrient digestibility, ammonia concentration, total VFA levels, and microbial protein synthesis. The diet containing 14% DM dietary protein and 70% DM energy, which contained 55%, 60%, or 65% CP RDP optimally increased nutrient digestibility, ammonia concentration, total VFA levels, and microbial protein synthesis. Thus, feed based on these RDP:RUP ratios can optimize ruminant productivity.

## Introduction

Ruminant feeds must be based on the degradability of feed ingredients, especially protein since it is used by both the host animals and rumen microorganisms. Microbes require ammonia (NH_3_) from protein degradation to form protein components of the cell wall. Ruminants require a true protein (bypass protein) and a microbial crude protein (CP) [1]. Thus, feeding a CP-based diet could be ineffective in terms of ruminant productivity.

In ruminants, proteins can be divided into two types: Rumen degradable protein (RDP) and rumen undegradable protein (RUP). RDP is degraded by enzymes secreted by ruminal bacteria, such as protease, peptidase, and deaminase, and is turned into peptides, amino acids, and NH_3_. NH_3_ is then converted into microbial CP (MCP), which flows in the liquid and solid phases of digesta to be absorbed as amino acids and peptides in the intestine, thus providing 50%-80% of the absorbable true protein [2,3]. RUP is another true protein that is not degraded by rumen microbes, instead flows directly to the abomasum and small intestine for direct use by the host. RUP is digested in the small intestine, where approximately 80% is absorbed as amino acids with MCP for tissue utilization. RUP is important for providing high-quality amino acids to highly productive ruminants compared to MCP [4].

Ruminant protein has three major functions: (i) To meet the RDP requirements of rumen microbes for maximum carbohydrate digestion and maximal microbial protein synthesis; (ii) to provide the protein needed for host animal maintenance, growth, optimal health, and reproduction with minimal RUP intake; and (iii) to fulfill the amino acid requirements of highly productive ruminants using minimal dietary CP [1]. Highly productive ruminants require a higher percentage of RUP in their diets to meet the amino acid requirements of the post-ruminal stage [5].

Efficient ruminant productivity requires optimal protein, energy levels, and RDP:RUP ratios in feed. Animal productivity can be increased by synchronizing the ruminal availability of carbohydrates and proteins [6]; whereas, non-synchronized protein and energy levels in feeds can reduce microbe protein synthesis. Furthermore, a low RDP level can decrease ruminal NH_3_-N levels, dry matter (DM) intake, and MCP. Excessive RDP will most likely be degraded to NH_3_-N, which is absorbed into the blood, then converted to urea in the liver before being excreted in the urine [7].

In previous studies, increased RDP levels in ruminant diets significantly increased nutrient digestibility, rumen fermentation, and microbial protein synthesis [8,9]; however, there has been a lack of research on these protein fractions in the diet of ruminants in Indonesia. Thus, we aimed to determine the optimum RDP:RUP ratio in ruminant diets, using a ruminant feed commonly used for cattle in Indonesia, and determined the resulting nutrient digestibility, rumen fermentation, and microbial protein synthesis.

## Materials and Methods

### Ethical approval

This research did not use any live animals so, ethical approval is not needed.

### Study period and location

This study was conducted from November 2019 to March 2020 at Ruminant Laboratory of Animal Science Faculty of Andalas University.

### Sample preparation and experimental diets

**Figure-1:**
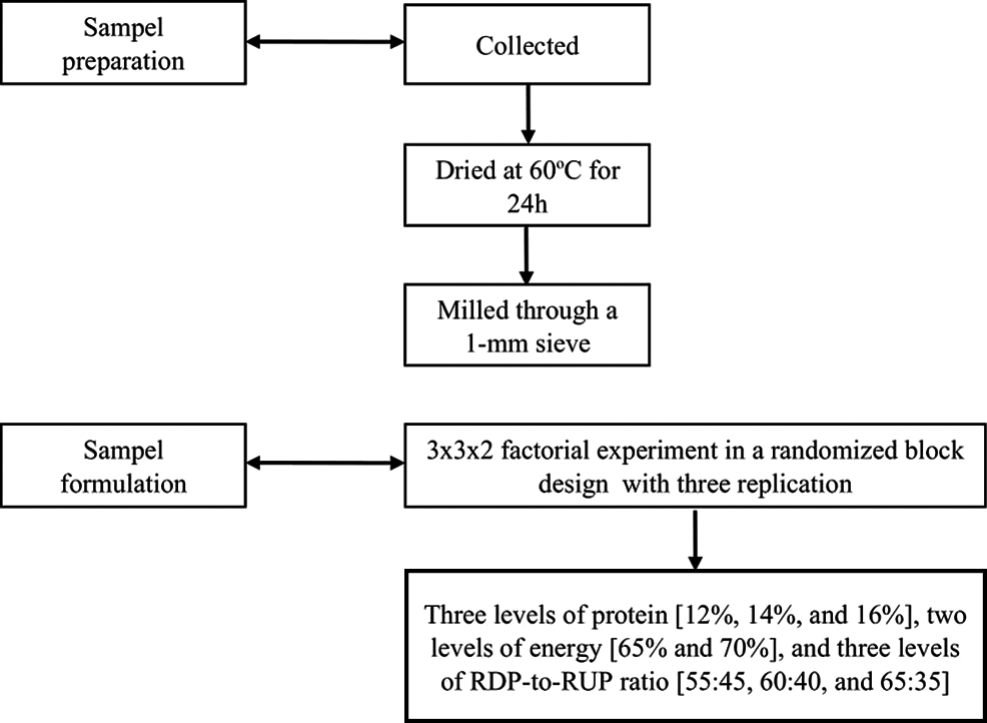
Flow diagram of sample preparation and formulation.

The plant species samples (*Pennisetum purpureum, Gliricidia sepium*, and *Indigofera zollingeriana*) were collected and identified by the authors from the UPT Teaching Farm, Faculty of Animal Science, Andalas University, Padang, Indonesia. The samples were dried at 60°C for 24 h in a forced-air oven, and then milled through a 1 mm sieve. Cassava (*Manihot esculenta*), maize (*Zea mays*), palm kernel cake (palm oil or *Elaeis guineensis*), rice bran (paddy or *Oryza sativa*), and tofu waste were obtained from a poultry shop. The chemical analysis included proximate analysis, Van Soest analysis, and the determination of the RDP and RUP levels in each sample [10]. The samples were then formulated into rations based on protein, energy levels, and RDP:RUP ratio. The experiment used a 3×3×2 factorial, randomized block design with three replications. Treatments consisted of three levels of protein (12%, 14%, and 16%), two levels of energy (65% and 70%), and three levels of RDP:RUP ratio (55:45, 60:40, and 65:35). The chemical composition of each treatment diet is given in Tables-1-3. The flow diagram of sample preparation and formulation is given in Figure-1.

**Table-1:** Chemical composition diet for protein 12% DM.

Component	65% THN (DM)			70% THN (DM)		
	
	RDP55	RDP60	RDP65	RDP55	RDP60	RDP65
Ingredient composition (%)						
Elephant grass (*Pennisetum purpureum*)	30	30	30	30	30	30
*Leucaena leucocephala*	6	8	3	6	12	3
*Indigofera zollingeriana*	2	4	8	2	2	9
Cassava (*Manihot esculenta*)	12	15	24	12	31	21
Palm kernel cake (*Elaeis guineensis*)	30	11	5	23	9	2
Maize (*Zea mays*)	8	4	2	20	8	18
Rice bran (*Oryza sativa*)	9	23	11	2	2	10
Tofu waste	2	4	15	4	5	6
Mineral	1	1	1	1	1	1
	100	100	100	100	100	100
Chemical composition (% DM)						
DM	89.37	89.36	88.91	88.62	89.32	88.37
RDP (%CP)	56.00	62.40	64.56	56.82	60.13	64.26
RUP (%CP)	43.00	36.60	33.44	42.18	38.87	34.74
Organic matter	92.89	91.59	91.72	93.79	93.42	93.01
CP	13.27	13.22	13.38	13.19	12.22	12.90
Crude fiber	21.17	21.08	19.57	18.34	16.95	16.90
NDF	22.59	23.71	23.00	22.59	24.56	23.23
ADF	13.96	14.88	14.45	13.96	15.48	14.66
Crude fat	4.85	4.79	4.27	4.30	3.18	3.61
Nitrogen Free Extract	54.03	52.19	53.98	58.16	59.38	59.75
TDN	67.04	66.76	67.71	68.52	69.21	68.93
Tannin	0.05	0.08	0.14	0.05	0.06	0.16

TDN=Total digestible nutrient, RDP=Rumen degradable protein, RUP=Rumen undegradable protein, NDF=Neutral detergent fiber, ADF=Acid detergent fiber, CP=Crude protein, DM=Dry matter

**Table-2:** Chemical composition diet for protein 14% DM.

Component	65% THN (DM)			70% THN (DM)		
	
	RDP55	RDP60	RDP65	RDP55	RDP60	RDP65
Ingredient composition (%)						
Elephant grass (*Pennisetum purpureum*)	30	30	30	30	30	30
*Leucaena leucocephala*	11	13	3	8	11	3
*Indigofera zollingeriana*	8	5	16	2	7	11
Cassava (*Manihot esculenta*)	8	7	10	9	23	21
Palm kernel cake (*Elaeis guineensis*)	27	10	3	26	15	2
Maize (*Zea mays*)	11	7	5	18	5	5
Rice bran (*Oryza sativa*)	2	22	24	2	2	9
Tofu waste	2	5	8	4	6	18
Mineral	1	1	1	1	1	1
	100	100	100	100	100	100
Chemical composition (% DM)						
DM	89.25	89.29	89.20	88.83	89.61	89.69
RDP (%CP)	55.96	62	66.86	55.89	59.55	66.30
RUP (%CP)	43.04	37	32.14	43.11	39.45	32.70
Organic matter	92.74	91.29	90.96	93.57	92.92	92.69
CP	15.38	14.80	15.39	13.82	14.00	14.50
Crude fiber	19.90	22.16	21.47	19.17	18.50	19.25
NDF	25.63	25.59	24.85	23.25	25.40	23.69
ADF	16.47	16.35	16.11	14.47	16.26	15.07
Crude fat	4.48	5.02	4.85	4.53	3.75	4.35
Nitrogen-free extract	53.42	49.68	49.45	56.27	56.78	53.85
TDN	67.25	66.59	66.55	68.13	68.29	68.73
Tannin	0.16	0.11	0.27	0.05	0.14	0.19

TDN=Total digestible nutrient, RDP=Rumen degradable protein, RUP=Rumen undegradable protein, NDF=Neutral detergent fiber, ADF=Acid detergent fiber, NFE=Nitrogen-free extract, CP=Crude protein, DM=Dry matter

**Table-3:** Chemical composition diet for protein 16% DM.

Component	65% THN (DM)			70% THN (DM)		
	
	RDP55	RDP60	RDP65	RDP55	RDP60	RDP65
Ingredient composition (%)						
Elephant grass (*Pennisetum purpureum*)	30	30	30	30	30	30
*Leucaena leucocephala*	14	5	2	17	14	3
*Indigofera zollingeriana*	8	15	18	3	4	13
Cassava (*Manihot esculenta*)	5	5	9	4	8	9
Palm kernel cake (*Elaeis guineensis*)	28	21	3	22	9	2
Maize (*Zea mays*)	5	2	4	17	15	5
Rice bran (*Oryza sativa*)	6	16	20	2	2	9
Tofu waste	3	5	13	4	17	28
Mineral	1	1	1	1	1	1
	100	100	100	100	100	100
Chemical composition (% DM)						
DM	89.72	89.64	89.47	89.00	89.39	90.10
RDP (%CP)	56.06	61.04	67.45	55.86	60.41	67.67
RUP (%CP)	42.94	37.96	31.55	43.14	37.83	31.33
Organic matter	92.07	91.29	91.18	92.90	93.16	92.48
CP	16.18	16.23	16.21	15.54	15.80	16.79
Crude fiber	21.69	22.58	21.47	19.60	19.33	21.19
NDF	26.61	25.28	24.99	26.44	25.68	24.16
ADF	17.23	16.41	16.28	16.95	16.40	15.49
Crude fat	4.91	5.20	4.99	4.54	4.68	5.37
Nitrogen free extract	49.71	47.65	48.30	53.49	48.28	47.66
TDN	66.49	65.86	66.86	67.82	68.98	68.55
Tannin	0.16	0.26	0.30	0.09	0.10	0.22

TDN=Total digestible nutrient, RDP=Rumen degradable protein, RUP=Rumen undegradable protein, NDF=Neutral detergent fiber, ADF=Acid detergent fiber, NFE=Nitrogen-free extract, CP=Crude protein, DM=Dry matter

### *In vitro* experiment

**Figure-2:**
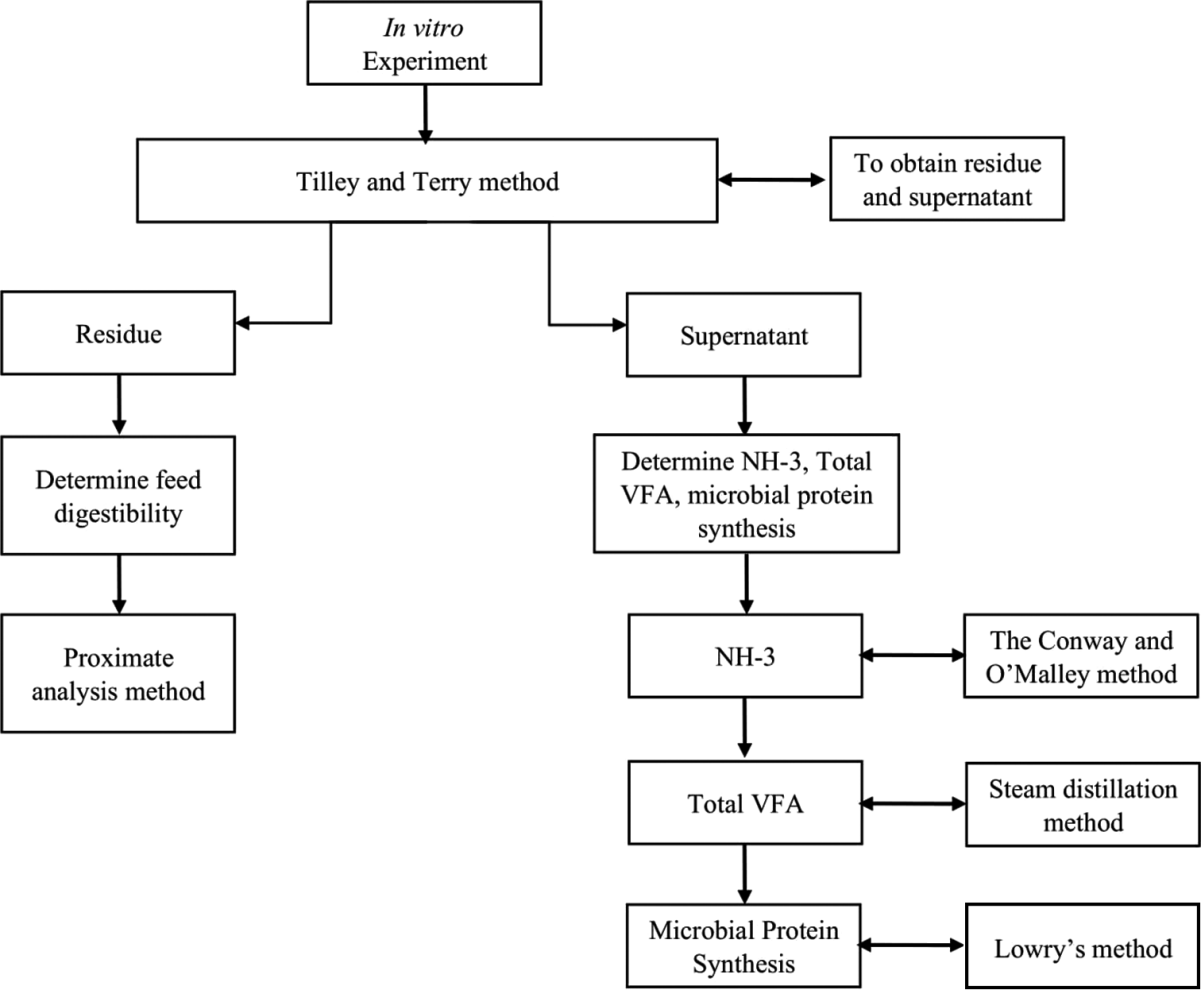
Flow diagram of the experimental methodology.

The flow diagram of the experimental methodology is given in Figure 2. An *in vitro* experiment was performed using the Tilley and Terry method [11], to determine feed digestibility, rumen fermentation characteristics, and microbial protein synthesis. In this experiment, rumen liquor was obtained from a slaughterhouse from three Pesisir cattle with an average BW ± 150 kg that were fed a diet of elephant grass and concentrate. Fresh rumen liquor was filtered using nylon (100 mm sieve size) and filled into pre-warmed (39°C) thermos flasks. Filtered rumen liquor was diluted with the buffer solution suggested by McDougall [12], at a ratio of 1:4 (rumen fluid:buffer solution). A 2.5 g sample was then incubated in each Erlenmeyer flask with 250 mL of mixed solution (rumen fluid and buffer) anaerobically by pumping CO_2_ gas into the flask, then sealing it with a rubber lid. Each flask was placed in a shaking incubator at a temperature of 39°C, and a rotational speed of 100 rpm for 48 h. After incubation, microbial activity was stopped by immersing the flask in ice water, after which the pH was measured. Thereafter, the supernatant was separated by placing the content of each flask in centrifuge tubes at 3000 rpm for 5 min at 4°C. The resulting supernatant was stored in bottles in a freezer at -18°C until NH_3_ and total volatile fatty acid (VFA) analysis could be completed. The NH_3_ levels were determined using the Conway and O’Malley method [13]. The total VFA levels were determined through steam distillation [14]. Microbial protein synthesis was determined using Lowry’s method [15]. The residue was filtered using Whatman No. 41 filter paper, and then dried in an oven at 60°C for 24 h [16]. This residue was analyzed using the Kjeldahl method to determine RUP digestibility. Subsequently, the feed digestibility was analyzed using the proximate analysis method [16]. A residue of 0.5 g was added to 40 ml 2% pepsin-HCl and further incubated for 24 h to determine RUP digestibility [11].

### Statistical analysis

This study was conducted using a factorial randomized block design. The obtained data were statistically analyzed using analysis of variances with Statistical Package for the Social Sciences (SPSS) software (IBM SPSS Statistics, USA) version 21.0 [17]. Data groups that showed a statistical significance (p<0.05) were further analyzed using least significant difference tests.

## Results

### Nutrient digestibility

An increase in protein, energy, and RDP levels increased nutrient digestibility (p<0.05). They also increased DM digestibility from 58.94% to 75.61%, organic matter digestibility from 60.13% to 76.97%, and CP digestibility from 42.71% to 64.95%. Rations with 16% protein lowered nutrient digestibility (p<0.05), and tended to decrease DM, organic matter, and CP digestibility. The digestibility of RUP in this experiment remained rather constant from 48.61% to 64.41%. *In vitro* DM, organic matter, CP, and RUP digestibility are presented in Table-4.

**Table-4:** Nutrient digestibility of experimental diets.

Factorial experiment			Variables			
	
Protein level (% DM)	Energy level (% DM)	RDP level (% CP)	DMD (%)	OMD (%)	CPD (%)	RUPD (%)
12	65	55	58.94^g^	60.13^k^	42.71^k^	60.69^c,d,e^
		60	59.01^g^	67.12^h^	50.01^i^	55.27^h^
		65	66.18^e^	62.58^j^	46.35^j^	56.01^g,h^
	70	55	69.17^d^	69.45^f^	55.00^h^	59.99^c,d,e,f^
		60	74.607^a,b^	74.49^c,d^	56.06^f,g,h^	51.77^i^
		65	74.24^b^	74.97^c,d^	57.35^d,e,f,g^	48.61^j^
14	65	55	73.97^b^	74.39^d^	67.73^a^	55.64^h^
		60	74.62^a,b^	75.04^c,d^	64.61^b^	60.21^c,d,e,f^
		65	74.41^a,b^	75.58^b,c^	60.8^c^	63.18^a,b^
	70	55	75.01^a,b^	75.46^b,c,d^	64.95^b^	62.34^a,b,c^
		60	75.29^a,b^	76.5^a,b^	61.85^c^	58.07^f,g^
		65	75.61^a^	76.97^a^	64.54^b^	53.81^h,i^
16	65	55	67.46^e^	67.96^g,h^	56.23^e,f,g,h^	58.49^e,f^
		60	66.75^e^	68.36^f,g^	58.49^d^	59.89^d,e,f^
		65	70.56^c^	72.19^e^	58.01^d,e^	64.41^a^
	70	55	69.03^d^	69.42^f^	57.81^d,e,f^	58.53^e,f^
		60	71.19^c^	72.36^e^	57.44^d,e,f,g^	60.16^c,d,e,f^
		65	63.05^f^	64.75^i^	55.60^g,h^	61.59^b,c,d^
SEM			0.73	0.68	0.87	0.57
P			0.05	0.05	0.05	0.05

^a,b,c,d,e,f,g,h,i,j,k^Superscript means significantly different in a column (p<0.05). DMD=Dry matter digestibility, OMD=Organic matter digestibility, CPD=Crude protein digestibility, RUP=Rumen undegradable protein digestibility, SEM=Standard error of mean

### Rumen fermentation characteristics

Increased protein, energy, and RDP levels did not affect pH, although there was a slight pH variation in the experimental diets (p>0.05). NH_3_ and total VFA tended to increase with increased protein, energy, and RDP levels (p<0.05). NH_3_ concentrations increased from 7.93 to 20.68 mM. Total VFA values increased from 93.33 to 151.67 mM (p<0.05). In contrast, 16% protein-rations tended to decrease NH_3_ concentrations from 11.05 to 9.07 mM and decrease total VFA from 116.67 to 101.67 mM (Table-5).

**Table-5:** Rumen fermentation characteristic and microbial protein synthesis of experimental diets.

Factorial experiment			Variables			
	
Protein level (% DM)	Energy level (% DM)	RDP level (% CP)	pH	NH3 (mg/100 mL)	Total VFA (mM)	MPS (mg/100 mL)
12	65	55	7.03	7.93^h^	93.33^i^	93.65^g^
		60	7.01	9.21^f,g,h^	96.67^h,i^	95.37^g^
		65	6.89	9.49^f,g^	103.33^g,h,i^	96.46^f,g^
	70	55	6.98	9.92^e,f,g^	113.33^e,f,g^	105.55^c,d^
		60	6.95	9.63^f,g^	110^f,g^	102.09^d,e^
		65	6.88	9.35^f,g^	106.67^f,g,h^	100.87^e,f^
14	65	55	6.95	11.62^d^	123.33^d,e^	109.53^c^
		60	7.00	13.03^c^	136.67^b,c^	114.40^b^
		65	6.96	11.05^d,e^	116.67^d,e,f^	108.91^c^
	70	55	6.95	17.28^b^	146.67^a,b^	138.01^a^
		60	6.98	19.83^a^	143.33^a,b^	137.09^a^
		65	6.92	20.68^a^	151.67^a^	139.25^a^
16	65	55	6.93	11.05^d,e^	116.67^d,e,f^	103.03^d,e^
		60	7.22	10.48^d,e,f^	126.67^c,d^	103.41^d,e^
		65	6.93	9.92^e,f,g^	106.67^f,g,h^	102.05^d,e^
	70	55	7.07	9.63^f,g^	103.33^g,h,i^	101.93^d,e^
		60	6.90	9.21^f,g,h^	113.33^e,f,g^	102.62^d,e^
		65	6.96	9.07^g,h^	101.67^g,h,i^	100.83^e,f^
SEM			0.04	0.51	2.46	1.95
p-value			0.05	0.05	0.05	0.05

^a,b,c,d,e,f,g,h,i,j^Superscript means significantly different in a column (p<0.05). NH_3_=Ammonia, VFA=Volatile fatty acid, MPS=Microbial protein synthesis, SEM=Standard error of mean

### Microbial protein synthesis

Increased protein, energy, and RDP levels significantly increased microbial protein synthesis (p<0.05) from 93.65 to 139.25 mg/100 mL. The 16% protein-rations tended to decrease microbial protein synthesis from 103.03 to 100.83 mg/100 mL (Table-5).

## Discussion

### Nutrient digestibility

The nutrient digestibility in this experiment increased with higher protein, energy, and RDP levels, indicating the beneficial effects of protein-energy synchronization and RDP levels on microbial protein synthesis. Nutrient digestibility correlated with the rumen microbial activity. An increase in the RDP level increased the availability of nitrogen for microbial protein synthesis, thus increasing microbe activity and their ability to digest feed. These results are in line with other studies that reported increased nutrient digestibility due to increased microbial activity as a result of increased RDP [18,19]. Conversely, an increase in RUP decreases NH_3_ levels and is a limiting factor in rumen microbial feed digestion activity. The previous studies have also reported that feed with high RUP levels decrease NH3 and reduces digestibility [7,20].

Increased nutrient digestibility indicates that the rumen is in better condition, leading to better fermentation. Better rumen fermentation and microbial activities lead to increased enzyme production, improved DM degradation, and decreased nutrient loss from the rumen. High digestibility improves ruminant productivity, because the nutrients can optimally utilize [5]. This improvement may also be due to the resulting nutrient abundance above what was required for improving digestibility. These results are in line with the previous studies [21,22], which stated that the availability of synchronized nutrient supply offered sufficient metabolic substrates for bacteria, which promoted their growth and increased nutrient digestibility. The ration with 16% protein levels tended to lower nutrient digestibility. We assumed that this ration could not reach an optimum protein-energy synchronization, which caused decreased microbial protein synthesis and nutrient digestibility.

Rumen undegraded protein digestibility (RUPD) is an important parameter in the updated protein evaluation systems for ruminant production and affects ruminant productivity. If RUP is indigestible, it supplies no metabolizable protein to the animal. In this experiment, RUPD varied among treatments but constantly ranged from 48% to 64%. These results concur with a previous study [23], which stated that the RUP digestibility varied considerably from 25% to 60%.

### Rumen fermentation characteristic

Rumen pH did not significantly change with the increases in protein, energy, and RDP levels and was in the range of 6.88-7.22. This value is within the normal range of 5.5-7 [24]. In a previous study, an increased dietary protein did not affect pH [25]. A decrease or increase in rumen pH disrupts the growth of rumen microbes, especially protozoa, which are highly sensitive to low rumen pH. As pH decreases, the energy normally used for the production of rumen microbial proteins is diverted to maintain a neutral pH in bacterial cells [26]. As RDP levels increased, rumen pH tended to decrease due to the tannins found in the legume used and the increased soluble carbohydrates in the diet. This is in line with the findings of a previous study [27], which reported that the addition of legumes tended to reduce rumen pH, although not significantly.

As expected, the major effects of altering dietary CP and RDP levels on the ruminal fermentation patterns were reflected in changes in the ruminal NH_3_-N level [28]. A previous study showed that an increase in the dietary protein level increased the NH_3_ level [25], which indicates that protein can be utilized by microbes in the form of NH_3_. Furthermore, increased NH_3_ levels in the rumen indicate high soluble protein levels and high DM digestibility in the diet [26]. RDP plays an important role in regulating rumen NH_3_ levels. The RDP:RUP ratio varies depending on how proteins are degraded and how nitrogen is absorbed and utilized by microbes [29]. RDP is utilized by rumen microbes as a source of nitrogen in microbial protein synthesis.

NH_3_ is a product of rumen microbial activity from digesting protein feed sources [27,28]. Rumen microbes, especially proteolytic bacteria, utilize RDP feed sources by secreting protease enzymes to convert proteins into peptides. Proteolytic bacteria secrete the enzyme peptidase, which converts peptides into amino acids. Furthermore, deaminase enzymes secreted by proteolytic bacteria convert amino acids into NH_3_, which plays a role in microbial protein synthesis [30,31]. A previous study reported that microbial protein synthesis increases NH_3_ production by 6-21 mMol [32]. In this study, 12% dietary protein levels increased NH_3_ from 7.93 to 9.92 mM, and the 14% dietary protein feed increased NH_3_ from 11.05 to 20.68 mM. Thus, it is likely that NH_3_ production could support microbial protein synthesis, indicating that the inclusion of legumes (*I. zollingeriana* and *Leucaena leucocephala*) provides organic matter for rumen microbial protein synthesis. This is in agreement with the findings of a previous study [33] that inclusion of *I. zollingeriana* at a high proportion provided sufficient organic material for rumen microbes, increased the fermentability profile, and increased the rate of rumen microbial protein synthesis.

In contrast, 16% dietary protein decreased NH_3_ levels from 11.05 to 9.07 mM. Thus, we assumed that microbial activity was not able to degrade the protein source into ammonia. As with microbial protein synthesis (Table-5), the microbial protein yield also decreased and affected the lower ammonia concentration in the rumen. Ammonia is an essential precursor for microbial protein synthesis in the rumen due to the inability of rumen microbes to directly transport amino acids into their cells [34]. Some other factors also affect the ammonia concentration in the rumen, such as protein fraction, rate of protein degradation, rate of passage, conversion efficiency of ammonia to microbial proteins, and clearance of ammonia from the rumen into the bloodstream [2].

Total VFA is a product of rumen microbial activity from digesting the energy source in the feed [25]. In this study, increased protein and RDP levels tended to increase the total VFA level. Specifically, the 12% dietary protein level increased the total VFA from 93.33 to 113.33 mM, and the 14% dietary protein increased the total VFA level from 123.33 to 151.67 mM. Thus, increased total VFA increased nutrient digestibility. As shown in Tables-4 and 5, nutrient digestibility tended to increase with total VFA, because one of the products of nutrient degradation is VFA. This finding agrees with those of a previous study [31].

The 16% dietary protein level decreased total VFA from 126.67 to 101.67 mM due to protein degradation and microbial protein synthesis. Decreased protein degradation and microbial protein synthesis decrease the production of total VFA, which is in line with Makmur *et al*. [35], who found that reduced degradation of feed proteins decreased VFAs and iso-VFAs production. It has also been reported that variation in RDP levels alters total VFA levels [9]. The degradation of feed sources by microbes produces ATPs, which would be used by the host, and VFA, which would be utilized by rumen microbes as a carbon source to form microbial proteins [8,36].

### Microbial protein synthesis

Microbial protein synthesis occurs due to the synchronization of protein feed sources and energy sources [21], which must be easily degradable. An increase in RDP can maximize microbial protein synthesis, but an increase in the dietary RUP level reduces microbial protein synthesis, which results in decreased digestibility [19,20]. In this study, increased proteins and RDP levels increased microbial protein synthesis, because of the availability of nitrogen from NH_3_ and C from total VFA. In the 12% dietary protein feed, microbial protein synthesis increased from 93.65 to 105.55 mg/100 mL, and the 14% dietary protein increased microbial protein synthesis from 108.91 to 139.25 mg/100 mL. The 16% dietary protein decreased microbial protein synthesis from 103.41 to 100.83 mg/100 mL. This indicates that protein-energy synchronization was not achieved. These findings agree with Lascano *et al*. [37] that efficient nutrient utilization and microbial protein synthesis can be achieved when ruminal reaction and protein and energy synchronization is optimal. Proteins are the most crucial source of nutrients for beef cattle, since they stimulate microbial protein synthesis and rumen fermentation, and improve productivity [38]. Increased microbial protein synthesis increases NH_3_ utilization and the effectiveness of fiber digestion, thus ensuring that the diet can be optimally used [26].

Approximately 50-80% of the amino acids absorbed are contributed from RDP to microbial protein synthesis [21,36]. It has also been reported that RDP can contribute as much as 100% to microbial protein synthesis in a forage-based or low-nutrient diet [38]. Proteins in poor-quality feed, in terms of amino acid profile and non-protein nitrogen, can be converted to high-quality proteins by rumen microbes. A major aspect of ruminant nutrients is the maximization of microbial growth and binding of RDP to microbial cells [39].

## Conclusion

The present study confirms that an increase in dietary protein (from 12% to 14% DM), energy (from 65% to 70% DM), and RDP (from 55% to 65% CP) increased nutrient digestibility, NH_3_ concentration, total VFA levels, and microbial protein synthesis. The diet containing 14% DM dietary protein and 70% DM energy contained RDP 55%, 60%, and 65% CP and is ideal to increase nutrient digestibility, NH_3_ concentration, total VFA levels, and microbial protein synthesis. These increases can reflect the benefit of RDP:RUP ration-based feeds to optimize the productivity of ruminants. Future research requires *in vivo* methods to determine the ideal RDP:RUP ratio in ruminant feeds.

## Authors’ Contributions

EMP, MZ, LW, and HH formulated the experimental design and experimental work at the laboratory. EMP drafted the manuscript and did data analysis under the guidance of MZ, LW, and HH. All authors read and approved the final manuscript.

## References

[1] Tedeschi L.O, Fox D.G, Fonseca M.A, Francis L, Cavalcanti L (2015). Models of protein and amino acid requirements for cattle. R. Bras. Zootec.

[2] Bach A, Calsamiglia S, Stern M.D (2005). Nitrogen metabolism in the rumen. J. Dairy. Sci.

[3] Kaufman J.D (2016). Effect of Varying Rumen Degradable and Undegradable Protein on Milk Production and Nitrogen Efficiency in Lactating Dairy Cows under Summer Conditions (Master of Science Degree). The University of Tennessee, Knoxville.

[4] Owens F.N, Qi S, Sapienza D.A (2014). Invited review:Applied protein nutrition of ruminants current status and future directions. Prof. Anim. Sci.

[5] Sharif M, Qamar H, Wahid A.A (2019). Effect of rumen degradable protein concentrations on nutrient digestibility, growth performance and blood metabolites in Beetal kids. Concepts Dairy Vet. Sci.

[6] Hall M.B (2013). Dietary starch source and protein degradability in diets containing sucrose:Effects on ruminal measures and proposed mechanism for degradable protein effects. J. Dairy. Sci.

[7] Bahrami-yekdangi M, Ghorbani G.R, Khorvash M, Khan M.A, Ghaffari M.H (2016). Reducing crude protein and rumen degradable protein with a constant concentration of rumen undegradable protein in the diet of dairy cows:Production performance, nutrient digestibility, nitrogen efficiency, and blood metabolites. J. Anim. Sci.

[8] Brooks M.A, Harvey R.M, Johnson N.F, Kerley M.S (2012). Rumen degradable protein supply effects microbial efficiency in continuous culture and growth in steers. J. Anim. Sci.

[9] Paula E.M, Monteiro H.F, Silva L.G, Benedeti P.D.B, Daniel J.L.P, Shenkoru T, Faciola A.P (2016). Effects of replacing soybean meal with canola meal differing in rumen-undegradable protein content on ruminal fermentation and gas production kinetics using 2 *in vitro* systems. J. Dairy Sci.

[10] Putri E.M, Zain M, Warly L, Hermon H (2019). *In vitro* evaluation of ruminant feed from West Sumatera based on chemical composition and content of rumen degradable and rumen undegradable proteins. Vet. World.

[11] Tilley J.M, Terry R.A (1963). A two-stage technique for *in vitro* digestion of forage crops. J. Br. Grassland Soc.

[12] McDougall E.I (1947). Studies on ruminant saliva. The composition and output of sheep's saliva. Biochem. J.

[13] Conway E.J, O'Malley E (1942). Microdiffusion methods. Ammonia and urea using buffered absorbents (revised methods for ranges greater than 10mg. N). Biochem. J.

[14] Prosedure G.L (1996). Departement of Dairy Science. University of Wisconsin Wisconsin.

[15] Lowry O.H, Rosebrough N.J, Farr A.L, Randall R.J (1951). Protein measurement with the Folin reagent. J. Biol. Chem.

[16] Association of Official Analytical. Official Methods of Analysis Association of Official Analytical Chemists International. Maryland, USA.

[17] SPSS. IBM SPSS Statistics for Windows (2012). Version 21.0. IBM Corp Armon, NY.

[18] Chandrasekharaiah M, Thulasi A, Suresh K.P, Sampath K.T (2011). Rumen degradable nitrogen requirements for optimum microbial protein synthesis and nutrient utilization in sheep fed on ﬁnger millet straw (*Eleucine coracana*) based diet. Anim. Feed Sci. Technol.

[19] Javaid A, Shahzad M.A, Nisa M, Sarwar M (2011). Ruminal dynamics of ad libitum feeding in buffalo bulls receiving different level of degradable protein. Livest. Sci.

[20] Akhtar M, Nisa M, Javais J (2017). Effect of varying levels of dietary rumen undegradable protein on dry matter intake, nutrient digestibility and growth performance of crossbred cattle heifers. Gomal Univ. J. Res.

[21] Hermon H, Suryahadi S, Wiryawan K.G, Hardjosoewignjo S (2008). Synchronized ratio of n-protein and energy supply in the rumen as a basis for ruminant animal ration formulation. Media Peternakan.

[22] Hao X.Y, Diaoa X.G, Yu S.C, Ding N, Mu C.T, Zhao J.X, Zhang J.X (2018). Nutrient digestibility, rumen microbial protein synthesis, and growth performance in sheep consuming rations containing sea buckthorn pomace. J. Anim. Sci.

[23] Buckner C.D, Klopfenstein T.J, Rolfe K.M, Griffin W.A, Lamothe M.J, Watson A.K, MacDonald J.C, Schacht W.H, Schroeder P (2013). Ruminally undegradable protein content and digestibility for forages using the mobile bag *in situ* technique. J. Anim. Sci.

[24] Puniya A.K, Singh R, Kamra D.N (2015). Rumen Microbiology:From Evolution to Revolution. Springer, New Delhi.

[25] Yang C, Bing-Wen S, Qi-Yu D, Hai J, Shu-Qin Z, Yan T (2016). Rumen fermentation and bacterial communities in weaned Chahaer lambs on diets with different protein levels. J. Integr. Agric.

[26] Uddin J.M, Haque K.Z, Jasimuddin K.M, Hasan K.M.M (2015). Dynamics of microbial protein synthesis in the rumen a review. Ann. Vet. Anim. Sci.

[27] Zain M, Putri E.M, Rusmana W.S.N, Erpomena Makmur M (2020). Effects of Supplementing *Gliricidia sepium* on ration based ammoniated rice straw in ruminant feed to decrease methane gas production and to improve nutrient digestibility (*in-vitro*). Int. J. Adv. Sci. Eng. Inf. Technol.

[28] Mutsvangwa T, Davies K.L, McKinnon J.J, Christensen D.A (2016). Effects of dietary crude protein and rumen-degradable protein concentrations on urea recycling, nitrogen balance, omasal nutrient flow, and milk production in dairy cows. J. Dairy. Sci.

[29] Tacoma R, Fields J, Ebenstein D.B, Lam Y.W, Greenwood S.L (2017). Ratio of dietary rumen degradable protein to rumen undegradable protein affects nitrogen partitioning but does not affect the bovine milk proteome produced by mid-lactation Holstein dairy cows. J. Dairy. Sci.

[30] Das L.K, Kundu S.S, Kumar D, Datt C (2014). Metabolizable protein systems in ruminant nutrition:A review. Vet. World.

[31] Ningrat R.W.S, Zain M, Erpomen Putri E.M, Makmur M (2019). Effects of *Leucaena leucocephala* supplementation to total mixed ration based on ammoniated rice straw on fiber digestibility and rumen fermentation characteristics *in vitro*. Int. J. Adv. Sci. Eng. Inf. Tech.

[32] McDonald P, Edwards R.A, Greenhalgh J.F.D, Morgan C.A, Sinclair L.A, Wilkinson R.G (2002). Animal Nutrition. Prentice Hall, London.

[33] Makmur M, Zain M, Agustin F, Sriagtula R, Putri E.M (2020a). *In vitro* rumen biohydrogenation of unsaturated fatty acids in tropical grass-legume rations. Vet. World.

[34] Jayanegara A, Novandri B, Yantina N, Ridla M (2017). Use of black soldier fly larvae (*Hermetia illucens*) to substitute soybean meal in ruminant diet:An *in vitro* rumen fermentation study. Vet. World.

[35] Makmur M, Zain M, Marlida Y, Khasrad K, Jayanegara A (2020b). *In vitro* ruminal biohydrogenation of C18 fatty acids in mixtures of *Indigofera zollingeriana* and *Brachiaria decumbens*. J. Indones. Trop. Anim. Agric.

[36] Hackmann T.J, Firkins J.L (2015). Maximizing efficiency of rumen microbial protein production. Front. Microbiol.

[37] Lascano G.J, Koch L.E, Heinrichs A.J (2016). Precision-feeding dairy heifers a high rumen-degradable protein diet with different proportions of dietary fiber and forage-to-concentrate ratios. J. Dairy Sci.

[38] Wanapat M (2009). Potential uses of local resources for ruminants. Trop. Anim. Health Prod.

[39] Givens D.I, Owen E, Adesogan A.T (2000). Current procedures, future requirements and the need for standardization. In:Forage Evaluation in Ruminant Nutrition. Cabi Publishing, Wallingford.

